# Post-colonoscopy Right Colon Perforation Misdiagnosed as Early Appendicitis: A Case Report

**DOI:** 10.7759/cureus.104779

**Published:** 2026-03-06

**Authors:** Srilekha Ravella, Oscar Castillo, Soshiant Raeesian, Sohrab Mardani, Joshua Simon, Khaled Saed

**Affiliations:** 1 Medicine, Burrell College of Osteopathic Medicine, Las Cruces, USA; 2 General Surgery, Delray Medical Center, Delray Beach, USA; 3 Surgery, Larkin Community Hospital, South Miami, USA

**Keywords:** appendicitis mimic, colonoscopy complication, iatrogenic complication, right colon perforation, right-sided hemicolectomy

## Abstract

Colon perforation is a rare but life-threatening emergency that may result from colonoscopy procedures, particularly in elderly patients with comorbidities. It often presents with non-specific abdominal pain that can mimic other acute abdominal conditions, including appendicitis. Early recognition and emergent repair are critical interventions needed to avoid adverse outcomes.

We present an 83-year-old man with a medical history of diabetes, chronic kidney disease, hypertension, and prior colonic polyps who presented to the emergency department with severe right lower quadrant pain two days post-colonoscopy. Abdominal computed tomography demonstrated findings typical of early appendicitis, but operative exploration revealed gangrenous cecal perforation and a retroperitoneal abscess. A laparoscopic-assisted right hemicolectomy with primary anastomosis, abscess drainage, and umbilical hernia repair were performed. Postoperative recovery was complicated by clinically significant anemia and mild basilar atelectasis but resolved with supportive management. This case emphasizes the possibility of iatrogenic colon perforations to mimic more common acute conditions such as appendicitis. It is important to maintain a high index of suspicion in elderly post-colonoscopy patients to allow for timely diagnosis and intervention in order to improve health outcomes.

## Introduction

Colonoscopy is one of the many modalities we use in both the diagnosis and treatment of colorectal disease. Colonoscopy is often indicated for the screening of colorectal cancer, the diagnosis of lower gastrointestinal diseases such as diverticulitis, inflammatory bowel disease, and polyps, and the treatment of polyps, bleeding, wounds, and other conditions. 

Both diagnostic and therapeutic colonoscopies are associated with a low overall complication rate of 0.28% to 0.5%, though complication rates increase following therapeutic procedures, such as polypectomy, where thermal injury may occur [1]. These complications range from mild to severe, with perforation of the colon being among the most life-threatening, with mortality increasing significantly if diagnosis is delayed [2]. 

Colonic perforations typically present with non-specific abdominal symptoms that may consist of localized or diffuse abdominal pain, nausea, vomiting, inability to pass flatus or stool, abdominal distension, fevers, and chills [3]. As perforations occur anywhere along the colon, they may mimic other acute abdominal conditions, including appendicitis, especially when imaging is non-specific [4].

Management may be non-operative, endoscopic, or surgical depending on timing, size, and clinical stability. When identified within four hours post-colonoscopy and bowel prep is adequate, small iatrogenic perforations up to approximately 20 mm can be treated endoscopically using through-the-scope or over-the-scope clips, followed by close monitoring. Surgery is typically reserved for large defects, failed closure, feculent peritonitis, or significant pneumoperitoneum [1,5].

We present a case of a post-polypectomy right colon perforation in an elderly male patient, who initially presented with clinical and radiographic features of early appendicitis. This case highlights how delayed thermal injury after colonoscopic polypectomy can cause perforation that mimics other causes of acute abdominal pain, creating diagnostic and therapeutic challenges in managing iatrogenic colon perforations.

## Case presentation

An 83-year-old man with a medical history significant for type 2 diabetes mellitus, stage 3 chronic kidney disease, hypertension, and prior colonic polyps presented to the emergency department with right lower quadrant pain two days after undergoing colonoscopic polypectomy. The patient reported that the pain began one day prior to presentation and had progressively worsened. It was described as constant and non-radiating and rated 9/10 in severity. The patient endorsed chills but denied fever, nausea, or vomiting.

On arrival, vital signs were notable for stage 2 hypertension (142/100 mmHg), while the patient was otherwise afebrile and hemodynamically stable. A focused physical examination demonstrated abdominal distension with right lower quadrant tenderness, rebound, and a positive McBurney sign. A small, reducible umbilical hernia was also found. General surgery was consulted for further monitoring and management.

To exclude other etiologies of acute right lower quadrant pain and to establish surgical candidacy, laboratory studies and CT imaging were obtained. Laboratory test results on initial presentation to the ED (Table 1) revealed leukocytosis with a white blood cell count of 17.5 × 10³/µL, hemoglobin of 12.3 g/dL, hematocrit of 37.3%, prothrombin time of 12.8 seconds, and an elevated serum glucose level of 222 mg/dL.

**Table 1:** Laboratory test results on initial presentation to the ED.

Laboratory Test	Patient Value	Reference Range
White Blood Cell Count	17.5 ×10³ /µL	4.0–11.0 /µL
Hemoglobin	12.3 g/dL	13.5–17.5 g/dL (Male)
Hematocrit	37.3%	41–53% (Male)
Prothrombin Time	12.8 Seconds	11–13.5 Seconds
Serum Glucose	222 mg/dL	70–99 mg/dL (Fasting)

**Figure 1:**
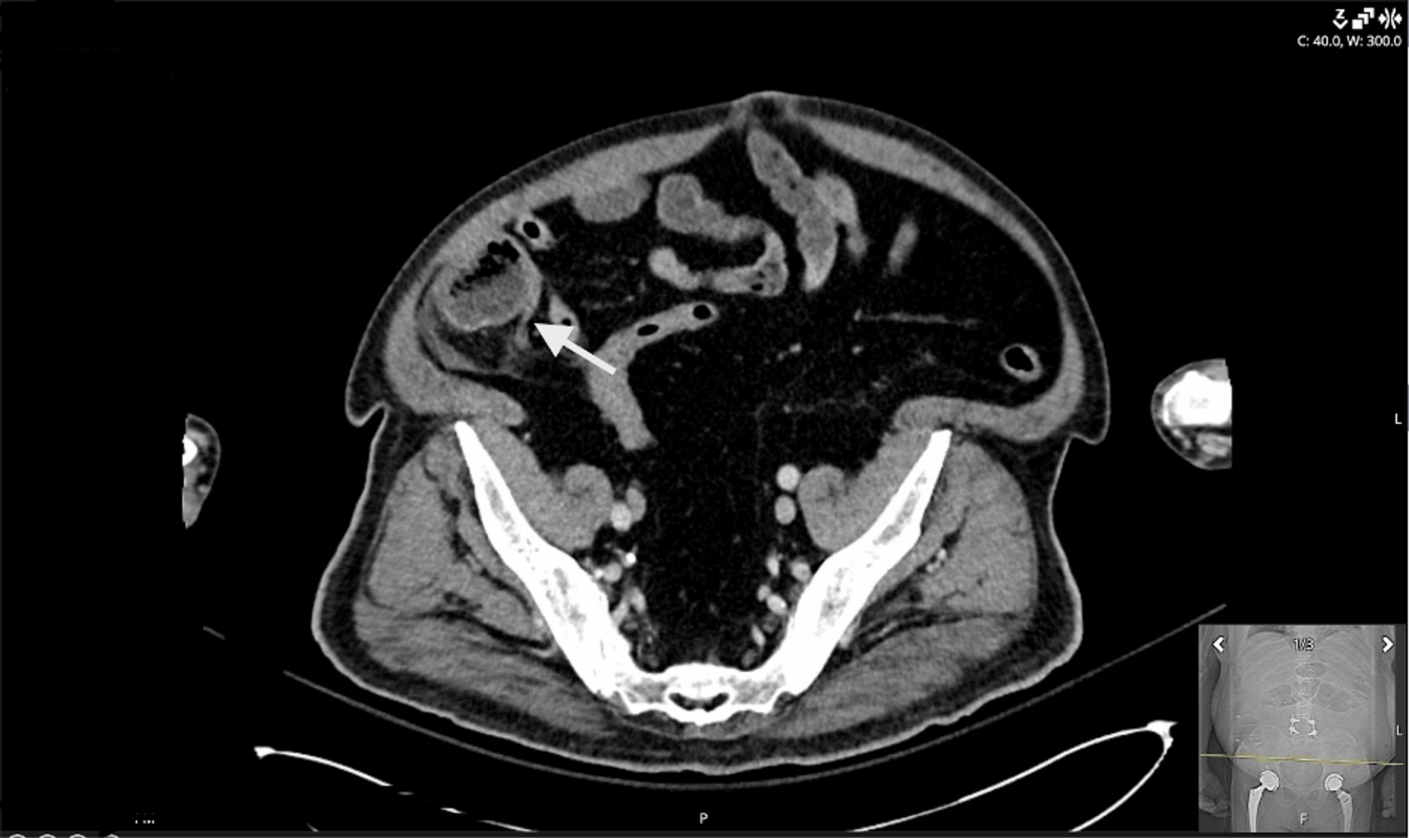
Contrast-enhanced axial CT of the pelvis demonstrating nonspecific right lower quadrant fat stranding adjacent to the appendiceal tip (white arrow).

Contrast-enhanced CT of the abdomen and pelvis (Figure 1) demonstrated a nondistended appendix with periappendiceal fat stranding, suspicious for early appendicitis. Subtle extraluminal air was identified posterolateral to the cecum, which was initially interpreted to be possibly related to appendiceal perforation.

Based on the patient’s clinical presentation and CT imaging results, a presumptive diagnosis of appendicitis was made, and a laparoscopic appendectomy was scheduled.

Intraoperatively, laparoscopy revealed a posterolateral cecal perforation with an anterolateral gangrenous patch, a retroperitoneal abscess, and an umbilical hernia. There was no evidence of appendiceal perforation or inflammation. 

To treat the findings above, a laparoscopic-assisted right hemicolectomy with primary ileocolic anastomosis, drainage of the abscess, primary umbilical hernia repair, and placement of a negative-pressure wound therapy device (wound vacuum-assisted closure (VAC)) were performed. The right colon was mobilized by a lateral-to-medial approach along the white line of Toldt using a LigaSure advanced bipolar vessel-sealing device. The right and middle colic vessels were ligated. Anastomosis was achieved with gastrointestinal anastomosis staplers and thoracoabdominal staplers and oversewn with 3-0 Vicryl. Estimated blood loss was 10 cc.

**Figure 2:**
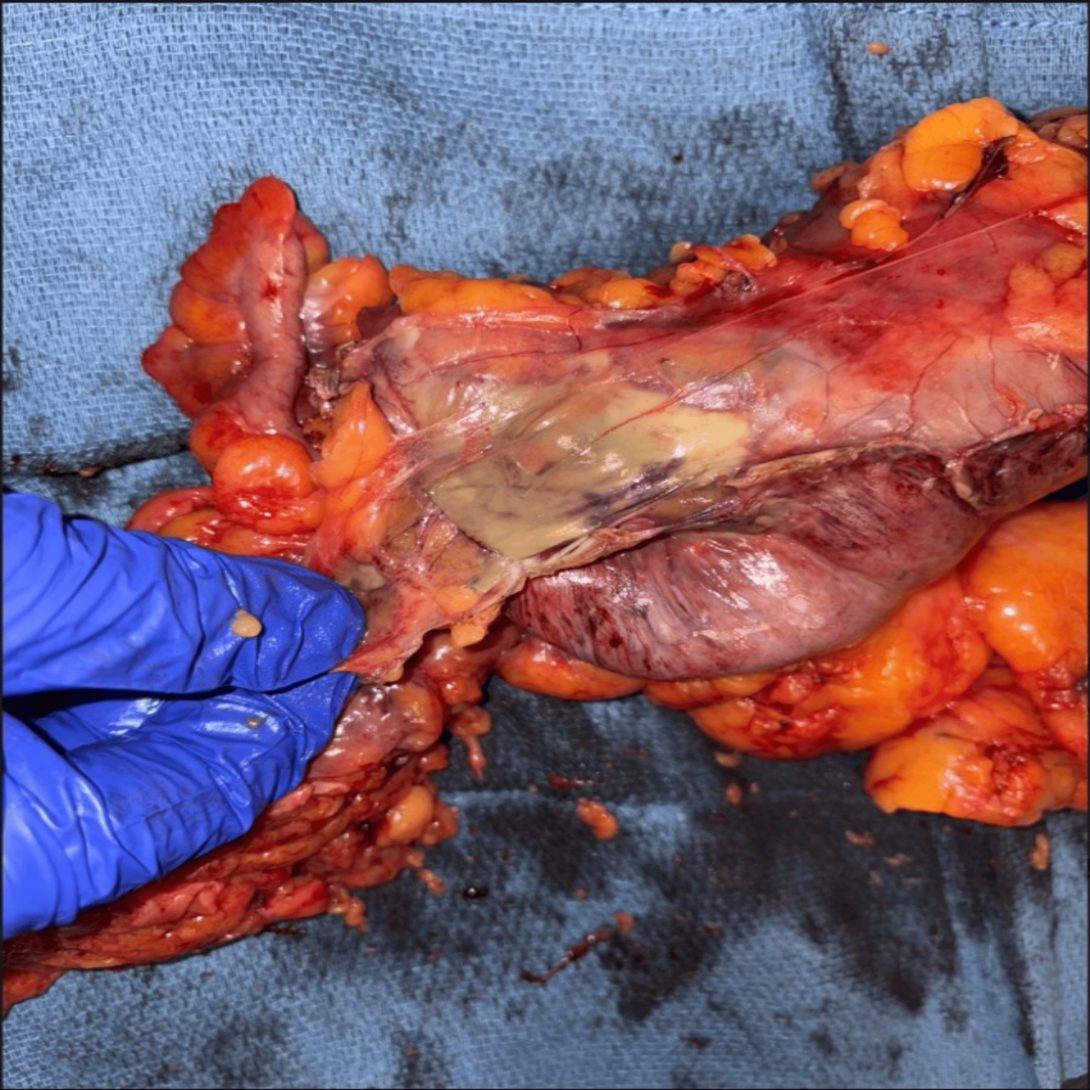
The resected gross specimen of the right colon demonstrates a site of transmural necrosis, with pale, ischemic discoloration seen throughout the colon and widespread inflammation suggesting injury secondary to thermal damage.

Pathologic findings of the resected specimen (Figure 2) revealed transmural necrosis and acute inflammation consistent with post-polypectomy thermal injury. Cultures identified normal mixed colonic flora without fungal or acid-fast bacilli growth.

Post-operatively, retrospective evaluation of pre-operative CT imaging confirmed that previously identified extraluminal air corresponded to the site of colon perforation rather than appendiceal perforation. On post-operative day (POD) 2, the patient reported right upper-quadrant pain and had not yet passed a bowel movement. Chest X-ray demonstrated basilar infiltrates and peribronchial thickening without pneumothorax. Laboratory trends showed significant improvement in leukocytosis and a mild drop in hemoglobin. On POD 5, laboratory test results (Table 2) revealed a white blood cell count of 6.4 ×10³ /µL, prothrombin time of 11.5 seconds, serum glucose of 151 mg/dL, hematocrit of 26.7%, and hemoglobin of 9.1 g/dL with positive stool occult blood. The mild drop in hemoglobin and positive stool occult blood were thought to be secondary to minor post-operative bleeding at the anastomotic site. The patient was managed with incentive spirometry, ambulation, intravenous piperacillin-tazobactam, and a bowel regimen. By POD 7, the patient tolerated a regular diet, remained hemodynamically stable, and was discharged home with outpatient follow-up.

**Table 2:** Laboratory test results on POD 5 POD: Post-operative day

Laboratory Test	Patient Value	Reference Range
White Blood Cell Count	6.4 ×10³ /µL	4.0–11.0 /µL
Hemoglobin	9.1 g/dL	13.5–17.5 g/dL (Male)
Hematocrit	26.7 %	41–53% (Male)
Prothrombin Time	11.5 Seconds	11–13.5 Seconds
Serum Glucose	151 mg/dL	70–99 mg/dL (Fasting)
Stool Occult Blood	Positive	Negative

## Discussion

Post-colonoscopy colonic perforation is a rare yet emergent iatrogenic complication that occurs in only 0.3%-3% of therapeutic procedures [6]. Post-polypectomy perforations usually occur in the rectosigmoid or cecal regions of the colon, making right-sided perforations extremely rare, often leading to diagnostic difficulties and delayed treatment [7].

In this case, an elderly male patient with multiple comorbidities presented two days after a colonoscopic polypectomy with clinical features suggestive of acute appendicitis. CT imaging demonstrated periappendiceal fat stranding with adjacent pneumoperitoneum without pericolic or diffuse fat stranding, which led to the initial misdiagnosis of appendicitis. However, intraoperative findings confirmed a cecal perforation with gangrene and retroperitoneal abscess, which are consistent with a post-polypectomy perforation.

The indication for surgical intervention in this case was not based solely on indeterminate imaging but rather on definitive intraoperative findings of cecal necrosis and a retroperitoneal abscess. This case underscores the diagnostic challenge of delayed thermal injury following polypectomy, where early imaging findings may be subtle or misleading. It highlights the importance of integrating recent procedural history and clinical presentation with imaging findings, as clinical suspicion should take precedence when evaluating acute abdominal pain after colonoscopy [5,8]. The use of a laparoscopic-assisted approach with wound VAC placement in an octogenarian demonstrates that emergent surgical management can be safely performed when guided by careful intraoperative judgment.

Post-polypectomy perforation is most often caused by thermal injury during electrocautery, which can lead to delayed transmural necrosis rather than immediate perforation [9]. The cecum is particularly vulnerable due to its thin wall and higher intraluminal pressures [10]. Elderly patients are at increased risk because of decreased tissue resilience and the presence of comorbidities such as diabetes and chronic kidney disease, which impair healing and immune response [11].

Radiographic diagnosis can be difficult in early or contained perforations, as CT findings may be subtle and non-specific. Limited extraluminal air or localized fat stranding can be mistaken for appendicitis or other inflammatory processes, as occurred in this case [12]. This highlights the importance of correlating imaging with clinical history, especially recent endoscopic procedures.

Management depends on the timing of diagnosis, perforation size, and patient stability. While small, early detected colon perforations may be treated conservatively or endoscopically, delayed presentations or those with abscess formation and necrosis typically require surgical intervention [13]. In elderly patients, prompt operative management for colon perforation is often necessary to prevent progression to sepsis and reduce morbidity.

This case highlights the importance of maintaining a high index of suspicion for iatrogenic injuries following endoscopic interventions, even when radiographic imaging may suggest otherwise. Such consideration is particularly essential in patients who are elderly or have multiple comorbidities.

## Conclusions

Iatrogenic colon perforation presents with non-specific abdominal symptoms that can mimic other more common acute abdominal conditions, including appendicitis. This case emphasizes the importance of maintaining a high index of suspicion for perforation in elderly, high-risk patients following endoscopic procedures. Early recognition and surgical intervention are critical to preventing morbidity and optimizing patient outcomes.
